# Dichlorido[μ-10,21-dimethyl-2,7,13,18-tetra­phenyl-3,6,14,17-tetra­aza­tricyclo­[17.3.1.1^8,12^]tetra­cosa-1(23),2,6,8,10,12(24),13,17,19,21-deca­ene-23,24-diolato]dicopper(II) ethanol hemisolvate dihydrate

**DOI:** 10.1107/S1600536810042248

**Published:** 2010-11-10

**Authors:** Sushil K. Gupta, Chanda Anjana, Ray J. Butcher, Neha Sen

**Affiliations:** a School of Studies in Chemistry, Jiwaji University, Gwalior 474 011, India; b Department of Chemistry, Howard University, 525 College Street NW, Washington, DC 20059, USA

## Abstract

The dinuclear title complex, [Cu_2_(C_46_H_38_N_4_O_2_)Cl_2_]·0.5C_2_H_5_OH·2H_2_O, is located on crystallographic inversion centres with two half-mol­ecules in the asymmetric unit. The two Cu^II^ atoms are coordinated by a hexa­dentate dianionic ligand formed *in situ* from the condensation of two tridentate ligands by four imine N atoms and two bridging phenolate O atoms along with two Cl atoms at axial positions. The coordination geometry around the metal atoms is distorted square-pyramidal (τ = 0.185 and 0.199). The non-bonding Cu⋯Cu distances are 2.9556 (12) and 2.9506 (12) Å in the two dimers. The packing is stabilized through solvent-mediated inter­molecular O—H⋯O and O—H⋯Cl hydrogen bonds. The diamine chain of one of the dimers is disordered over two positions in a 0.680 (5):0.320 (5) ratio.

## Related literature

For general background to phenol-based Schiff base metal complexes with *N*,*N*,*O* donors, see: Generex & Barton (2010 ▶); Fenton *et al.* (2010 ▶); Reisner *et al.* (2008 ▶). For related phenol-based Schiff base complexes, see: Gupta *et al.* (2002 ▶, 2007 ▶, 2008 ▶). For a description of the geometry of penta­coordinated atoms, see: Addison *et al.* (1984 ▶).
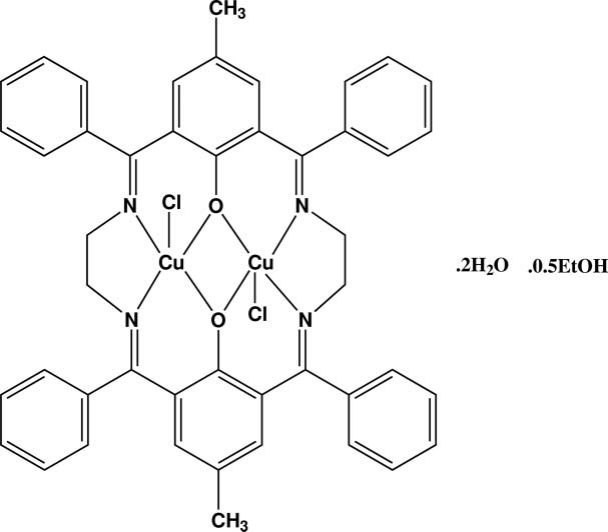


## Experimental

### 

#### Crystal data

[Cu_2_(C_46_H_38_N_4_O_2_)Cl_2_]·0.5C_2_H_6_O·2H_2_O
*M* *_r_* = 935.85
Monoclinic, 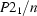

*a* = 15.1983 (4) Å
*b* = 16.2680 (5) Å
*c* = 19.3344 (5) Å
β = 103.964 (2)°
*V* = 4639.1 (2) Å^3^
*Z* = 2
Mo *K*α radiation
μ = 1.08 mm^−1^
*T* = 293 K
0.30 × 0.20 × 0.20 mm

#### Data collection

Bruker Kappa APEXII CCD diffractometer
Absorption correction: multi-scan (*SADABS*; Bruker, 1999 ▶) *T*_min_ = 0.738, *T*_max_ = 0.813
36333 measured reflections
6669 independent reflections
4664 reflections with *I* > 2σ(*I*)
*R*_int_ = 0.054
θ_max_ = 23.3°

#### Refinement

*R*[*F* ^2^ > 2σ(*F* ^2^)] = 0.044
*wR*(*F* ^2^) = 0.137
*S* = 1.05
6669 reflections
575 parameters
13 restraints
H atoms treated by a mixture of independent and constrained refinement
Δρ_max_ = 0.51 e Å^−3^
Δρ_min_ = −0.34 e Å^−3^

### 

Data collection: *APEX2* (Bruker, 2004 ▶); cell refinement: *SAINT-Plus* (Bruker, 2004 ▶); data reduction: *SAINT-Plus* and *XPREP* (Bruker, 2004 ▶); program(s) used to solve structure: *SHELXS97* (Sheldrick, 2008 ▶); program(s) used to refine structure: *SHELXL97* (Sheldrick, 2008 ▶); molecular graphics: *SHELXTL* (Sheldrick, 2008 ▶); software used to prepare material for publication: *SHELXTL*.

## Supplementary Material

Crystal structure: contains datablocks I, global. DOI: 10.1107/S1600536810042248/bt5381sup1.cif

Structure factors: contains datablocks I. DOI: 10.1107/S1600536810042248/bt5381Isup2.hkl

Additional supplementary materials:  crystallographic information; 3D view; checkCIF report

## Figures and Tables

**Table 1:** Hydrogen-bond geometry (Å, °)

*D*—H⋯*A*	*D*—H	H⋯*A*	*D*⋯*A*	*D*—H⋯*A*
O3—H4⋯O2*W*	0.82	2.20	2.96 (2)	154
O1*W*—H1*W*1⋯Cl1^i^	0.82 (2)	2.54 (2)	3.355 (4)	171 (4)
O1*W*—H1*W*2⋯Cl2	0.82 (2)	2.56 (2)	3.374 (4)	174 (3)
O2*W*—H2*W*1⋯O3*W*	0.85	1.86	2.71 (3)	179
O2*W*—H2*W*2⋯O3	0.97	2.27	2.96 (2)	127

Symmetry code: (i) 
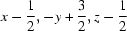
.

**Fig. 1.**
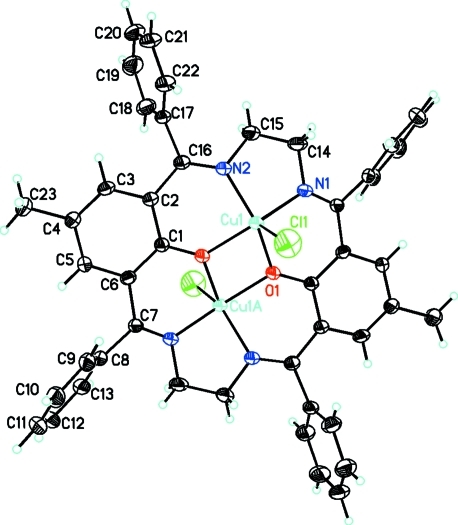
The molecular structure of the title compound with the atom numbering scheme. Displacement ellipsoides are drawn at 50° probability level. Only one of the molecule in the asymmetric unit is shown for clarity. Solvent molecules are omitted.

**Fig. 2.**
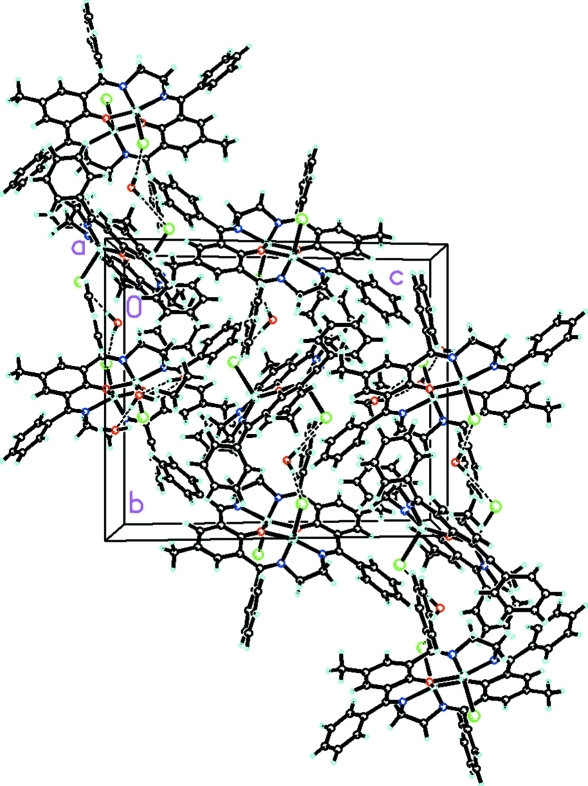
The packing diagram of the molecules in the unit cell viewed down the a axis. The intermolecular H-bonding interactions are shown with dashed lines.

## supplementary crystallographic information

### Comment

Phenol-based Schiff base metal complexes with NNO donors have recently drawn more attention due to their adjustable coordination ability and interesting application in bio-inorganic and therapeutic chemistry (Generex & Barton, 2010; Fenton *et al.*, 2010; Reisner *et al.*, 2008). As part of our investigations on the phenol based Schiff base complexes (Gupta *et al.*, 2002, 2007, 2008), we herein report the synthesis and crystal structure of a dimeric copper (II) complex. The molecular structure of the complex is shown in Fig.1. The dimeric complex consists of [Cu_2_Cl_2_{(µ-O)C_6_H_2_(Me-4)PhCN(CH _2_)_2_NCPh}_2_].2H_2_O. 0.5 EtOH wherein the copper (II) ion is five- coordinated with four imine N, two bridging phenolato O and two Cl at axial positions, giving a square pyramidal geometry [τ = 0.185 and 0.199 for Cu1 and Cu2, respectively (Addison *et al.*, 1984)]. The copper atoms are displaced from the basal planes by 0.430 (1) and 0.436 (1) Å, respectively. The average Cu—Cl, Cu—O and Cu—N bond lengths are 2.428 (2), 1.931 (13) and 1.927 (4) A, respectively. the non-bonding Cu···..Cu distances are 2.9556 (12) and 2.9506 (12) Å. The packing is stabilized through solvent mediated intermolecular hydrogen bonds.

### Experimental

Copper (II) chloride dihydrate (0.086 g, 0.51 mmol) dissolved in dry ethanol (20 ml) was added dropwise to an ethanol solution of tridentate ligand, 2-{*N*-(2-aminoethyl)benzimidoyl}-6-benzoyl-4-methylphenol (aebmpH) (0.365 g, 1.02 mmol) and heated to reflux. The clear solution so obtained was allowed to cool and dark green crystals suitable for X-ray diffraction were obtained in 40° yield.

### Refinement

The diamine chain of one of the dimers in the asymmetric unit is disordered. The site occupancy of the disordered components were refined [0.680 (5)/0.320 (5)]. The H atom bound to C atoms were restrained as riding atoms with d(C—H) = 0.93Å for aromatic CH and 0.97Å for secondary CH_2_ groups and 0.96Å for CH_3_ groups with *U*_iso_(H) = 1.2*U*_equ_(C). The hydroxyl hydrogen of ethanol solvate was fixed at 0.82 Å. The crystals did not scatter beyond 2θ of 46.7°. Recrystallization and fresh data collection did not improve the situation.

### Crystal data

**Table:**

[Cu_2_(C_46_H_38_N_4_O_2_)Cl_2_]·0.5C_2_H_6_O·2H_2_O	*F*(000) = 1932
*M**_r_* = 935.85	*D*_x_ = 1.340 Mg m^−^^3^
Monoclinic, *P*2_1_/*n*	Mo *K*α radiation, λ = 0.71073 Å
Hall symbol: -P 2yn	Cell parameters from 6395 reflections
*a* = 15.1983 (4) Å	θ = 2.1–22.5°
*b* = 16.2680 (5) Å	µ = 1.08 mm^−^^1^
*c* = 19.3344 (5) Å	*T* = 293 K
β = 103.964 (2)°	Needle, brown
*V* = 4639.1 (2) Å^3^	0.30 × 0.20 × 0.20 mm
*Z* = 2	

### Data collection

**Table:**

Bruker Kappa APEXII CCD diffractometer	6669 independent reflections
Radiation source: fine-focus sealed tube	4664 reflections with *I* > 2σ(*I*)
graphite	*R*_int_ = 0.054
ω and φ scan	θ_max_ = 23.3°, θ_min_ = 2.0°
Absorption correction: multi-scan (*SADABS*; Bruker, 1999)	*h* = −16→16
*T*_min_ = 0.738, *T*_max_ = 0.813	*k* = −18→18
36333 measured reflections	*l* = −21→21

### Refinement

**Table:**

Refinement on *F*^2^	Primary atom site location: structure-invariant direct methods
Least-squares matrix: full	Secondary atom site location: difference Fourier map
*R*[*F*^2^ > 2σ(*F*^2^)] = 0.044	Hydrogen site location: inferred from neighbouring sites
*wR*(*F*^2^) = 0.137	H atoms treated by a mixture of independent and constrained refinement
*S* = 1.05	*w* = 1/[σ^2^(*F*_o_^2^) + (0.0717*P*)^2^ + 3.5141*P*] where *P* = (*F*_o_^2^ + 2*F*_c_^2^)/3
6669 reflections	(Δ/σ)_max_ = 0.001
575 parameters	Δρ_max_ = 0.51 e Å^−^^3^
13 restraints	Δρ_min_ = −0.34 e Å^−^^3^

### Special details

**Table:**

Geometry. All e.s.d.'s (except the e.s.d. in the dihedral angle between two l.s. planes) are estimated using the full covariance matrix. The cell e.s.d.'s are taken into account individually in the estimation of e.s.d.'s in distances, angles and torsion angles; correlations between e.s.d.'s in cell parameters are only used when they are defined by crystal symmetry. An approximate (isotropic) treatment of cell e.s.d.'s is used for estimating e.s.d.'s involving l.s. planes.
Refinement. Refinement of *F*^2^ against ALL reflections. The weighted *R*-factor *wR* and goodness of fit *S* are based on *F*^2^, conventional *R*-factors *R* are based on *F*, with *F* set to zero for negative *F*^2^. The threshold expression of *F*^2^ > σ(*F*^2^) is used only for calculating *R*-factors(gt) *etc*. and is not relevant to the choice of reflections for refinement. *R*-factors based on *F*^2^ are statistically about twice as large as those based on *F*, and *R*- factors based on ALL data will be even larger.

### Fractional atomic coordinates and isotropic or equivalent isotropic displacement parameters (Å^2^)

**Table:**

	*x*	*y*	*z*	*U*_iso_*/*U*_eq_	Occ. (<1)
Cu1	1.09084 (3)	0.47211 (2)	1.039422 (19)	0.04010 (11)	
Cu2	0.52512 (3)	0.47246 (3)	0.57491 (2)	0.04566 (12)	
Cl1	1.17520 (9)	0.59912 (7)	1.06780 (7)	0.0984 (4)	
Cl2	0.53483 (9)	0.59915 (7)	0.64348 (7)	0.0943 (4)	
O1	0.97493 (14)	0.50362 (14)	1.05664 (11)	0.0473 (6)	
O2	0.58164 (14)	0.50367 (15)	0.49873 (11)	0.0519 (7)	
O3	0.5295 (5)	0.5394 (8)	0.7957 (6)	0.283 (7)	0.50
H4	0.5485	0.5341	0.8389	0.425*	0.50
O1W	0.5206 (3)	0.7508 (3)	0.5223 (2)	0.1308 (15)	
H1W1	0.5577 (17)	0.7868 (13)	0.538 (2)	0.196*	
H1W2	0.526 (3)	0.7165 (15)	0.5540 (15)	0.196*	
O2W	0.6367 (7)	0.4809 (11)	0.9357 (13)	0.370 (13)	0.50
H2W1	0.6163	0.4422	0.9569	0.555*	0.50
H2W2	0.6163	0.4649	0.8861	0.555*	0.50
O3W	0.5749 (10)	0.3569 (14)	1.0049 (11)	0.443 (12)	0.50
H3W1	0.5202	0.3669	0.9609	0.665*	0.50
H3W2	0.5644	0.3602	1.0442	0.665*	0.50
N1	1.12269 (17)	0.41850 (16)	1.13139 (13)	0.0396 (7)	
N2	1.16146 (18)	0.39153 (17)	1.00425 (13)	0.0458 (8)	
N3	0.63697 (17)	0.41998 (16)	0.62346 (13)	0.0435 (7)	
N4	0.46031 (18)	0.39075 (18)	0.61374 (15)	0.0554 (9)	
C1	1.04385 (19)	0.48096 (18)	0.88252 (15)	0.0341 (8)	
C2	1.1145 (2)	0.42521 (19)	0.87872 (15)	0.0369 (8)	
C3	1.1337 (2)	0.4126 (2)	0.81264 (16)	0.0443 (9)	
H3	1.1805	0.3768	0.8101	0.053*	
C4	1.0874 (2)	0.4498 (2)	0.75130 (16)	0.0446 (9)	
C5	1.0176 (2)	0.50157 (19)	0.75585 (16)	0.0392 (8)	
H5	0.9847	0.5265	0.7143	0.047*	
C6	0.99364 (19)	0.51866 (18)	0.81944 (15)	0.0350 (8)	
C7	0.9157 (2)	0.57477 (18)	0.81663 (15)	0.0362 (8)	
C8	0.8847 (2)	0.62559 (19)	0.75101 (15)	0.0400 (9)	
C9	0.9455 (3)	0.6785 (2)	0.73011 (16)	0.0494 (10)	
H9	1.0064	0.6783	0.7543	0.059*	
C10	0.9154 (3)	0.7317 (2)	0.67306 (19)	0.0651 (12)	
H10	0.9557	0.7680	0.6599	0.078*	
C11	0.8266 (3)	0.7308 (2)	0.63641 (19)	0.0720 (13)	
H11	0.8068	0.7667	0.5984	0.086*	
C12	0.7674 (3)	0.6784 (3)	0.65477 (19)	0.0695 (13)	
H12	0.7073	0.6778	0.6288	0.083*	
C13	0.7953 (2)	0.6251 (2)	0.71241 (17)	0.0513 (10)	
H13	0.7540	0.5893	0.7248	0.062*	
C14	1.2011 (2)	0.3657 (2)	1.13193 (18)	0.0608 (11)	
H14A	1.2563	0.3972	1.1486	0.073*	
H14B	1.2020	0.3207	1.1650	0.073*	
C15	1.1991 (3)	0.3324 (3)	1.06185 (18)	0.0741 (13)	
H15A	1.1627	0.2828	1.0546	0.089*	
H15B	1.2602	0.3177	1.0596	0.089*	
C16	1.1640 (2)	0.3759 (2)	0.93993 (16)	0.0393 (8)	
C17	1.2154 (2)	0.3023 (2)	0.92484 (16)	0.0430 (9)	
C18	1.1723 (3)	0.2301 (2)	0.90520 (18)	0.0561 (10)	
H18	1.1095	0.2274	0.8970	0.067*	
C19	1.2206 (3)	0.1607 (3)	0.8974 (2)	0.0750 (13)	
H19	1.1905	0.1112	0.8847	0.090*	
C20	1.3116 (3)	0.1643 (3)	0.9080 (2)	0.0838 (14)	
H20	1.3441	0.1172	0.9029	0.101*	
C21	1.3553 (3)	0.2362 (3)	0.9260 (2)	0.0807 (14)	
H21	1.4179	0.2387	0.9323	0.097*	
C22	1.3082 (2)	0.3061 (3)	0.93507 (19)	0.0614 (11)	
H22	1.3388	0.3554	0.9480	0.074*	
C23	1.1097 (3)	0.4335 (3)	0.68094 (18)	0.0719 (12)	
H23A	1.1262	0.3769	0.6785	0.108*	
H23B	1.1593	0.4679	0.6764	0.108*	
H23C	1.0576	0.4455	0.6430	0.108*	
C24	0.6667 (2)	0.52384 (19)	0.50571 (15)	0.0368 (8)	
C25	0.3096 (2)	0.41742 (19)	0.54154 (15)	0.0372 (8)	
C26	0.2183 (2)	0.39777 (19)	0.53150 (16)	0.0412 (9)	
H26	0.2023	0.3592	0.5618	0.049*	
C27	0.1505 (2)	0.4319 (2)	0.47948 (16)	0.0396 (8)	
C28	0.1757 (2)	0.48855 (19)	0.43462 (16)	0.0387 (8)	
H28	0.1305	0.5125	0.3992	0.046*	
C29	0.7350 (2)	0.48847 (18)	0.56032 (15)	0.0356 (8)	
C30	0.7164 (2)	0.42868 (19)	0.61303 (15)	0.0382 (8)	
C31	0.7943 (2)	0.37859 (19)	0.65308 (17)	0.0424 (9)	
C32	0.8411 (2)	0.3293 (2)	0.61612 (19)	0.0515 (10)	
H32	0.8268	0.3307	0.5666	0.062*	
C33	0.9089 (3)	0.2780 (2)	0.6522 (2)	0.0712 (13)	
H33	0.9389	0.2439	0.6268	0.085*	
C34	0.9321 (3)	0.2768 (3)	0.7248 (3)	0.0818 (15)	
H34	0.9781	0.2422	0.7489	0.098*	
C35	0.8880 (3)	0.3265 (3)	0.7621 (2)	0.0721 (13)	
H35	0.9044	0.3260	0.8117	0.087*	
C36	0.8191 (2)	0.3776 (2)	0.72694 (18)	0.0533 (10)	
H36	0.7893	0.4113	0.7528	0.064*	
C37	0.6168 (3)	0.3678 (4)	0.6819 (3)	0.0512 (14)	0.680 (5)
H37A	0.6141	0.4017	0.7226	0.061*	0.680 (5)
H37B	0.6638	0.3269	0.6970	0.061*	0.680 (5)
C38	0.5260 (3)	0.3264 (4)	0.6524 (3)	0.0598 (17)	0.680 (5)
H38A	0.5325	0.2828	0.6198	0.072*	0.680 (5)
H38B	0.5037	0.3026	0.6909	0.072*	0.680 (5)
C37'	0.6112 (7)	0.3476 (9)	0.6580 (6)	0.0512 (14)	0.320 (5)
H37C	0.6584	0.3347	0.7001	0.061*	0.320 (5)
H37D	0.6047	0.3011	0.6257	0.061*	0.320 (5)
C38'	0.5228 (7)	0.3623 (8)	0.6789 (7)	0.0598 (17)	0.320 (5)
H38C	0.5010	0.3119	0.6958	0.072*	0.320 (5)
H38D	0.5303	0.4036	0.7161	0.072*	0.320 (5)
C39	0.3754 (2)	0.3723 (2)	0.59562 (17)	0.0444 (9)	
C40	0.3429 (2)	0.2996 (2)	0.62927 (18)	0.0482 (9)	
C41	0.3327 (3)	0.2247 (2)	0.5969 (2)	0.0681 (12)	
H41	0.3441	0.2184	0.5520	0.082*	
C42	0.3054 (3)	0.1576 (3)	0.6310 (3)	0.0912 (17)	
H42	0.3008	0.1059	0.6098	0.109*	
C43	0.2855 (3)	0.1676 (3)	0.6957 (3)	0.0975 (16)	
H43	0.2652	0.1230	0.7176	0.117*	
C44	0.2948 (3)	0.2402 (3)	0.7275 (2)	0.0872 (15)	
H44	0.2820	0.2458	0.7719	0.105*	
C45	0.3234 (2)	0.3077 (3)	0.6954 (2)	0.0652 (12)	
H45	0.3296	0.3585	0.7181	0.078*	
C46	0.0538 (2)	0.4050 (2)	0.46937 (19)	0.0549 (10)	
H46A	0.0382	0.4044	0.5146	0.082*	
H46B	0.0465	0.3509	0.4490	0.082*	
H46C	0.0147	0.4426	0.4380	0.082*	
C47	0.3985 (7)	0.4819 (7)	0.8194 (8)	0.250 (11)	0.50
H47A	0.3347	0.4744	0.7997	0.375*	0.50
H47B	0.4088	0.4974	0.8686	0.375*	0.50
H47C	0.4298	0.4314	0.8157	0.375*	0.50
C48	0.4334 (9)	0.5483 (9)	0.7789 (9)	0.292 (9)	0.50
H48A	0.4084	0.5420	0.7280	0.350*	0.50
H48B	0.4167	0.6021	0.7932	0.350*	0.50

### Atomic displacement parameters (Å^2^)

**Table:**

	*U*^11^	*U*^22^	*U*^33^	*U*^12^	*U*^13^	*U*^23^
Cu1	0.0408 (2)	0.0455 (2)	0.03363 (19)	0.01844 (18)	0.00837 (16)	0.00292 (17)
Cu2	0.0359 (2)	0.0530 (2)	0.0464 (2)	−0.00232 (19)	0.00656 (18)	0.02143 (19)
Cl1	0.1143 (9)	0.0656 (7)	0.1098 (9)	−0.0300 (7)	0.0164 (8)	−0.0062 (7)
Cl2	0.1024 (9)	0.0662 (7)	0.1126 (9)	0.0039 (7)	0.0227 (8)	−0.0161 (7)
O1	0.0457 (12)	0.0634 (14)	0.0318 (11)	0.0272 (11)	0.0076 (10)	0.0027 (10)
O2	0.0329 (12)	0.0715 (15)	0.0484 (13)	−0.0072 (11)	0.0040 (10)	0.0273 (12)
O3	0.356 (14)	0.345 (15)	0.188 (9)	0.215 (12)	0.141 (10)	0.125 (9)
O1W	0.115 (3)	0.156 (3)	0.126 (3)	−0.016 (3)	0.037 (2)	0.023 (3)
O2W	0.065 (6)	0.325 (19)	0.71 (4)	0.021 (9)	0.071 (12)	0.06 (2)
O3W	0.179 (12)	0.61 (3)	0.51 (2)	0.054 (16)	0.029 (14)	−0.38 (2)
N1	0.0407 (14)	0.0414 (15)	0.0360 (13)	0.0166 (12)	0.0075 (12)	0.0054 (12)
N2	0.0478 (15)	0.0525 (16)	0.0367 (14)	0.0245 (13)	0.0094 (12)	0.0040 (12)
N3	0.0389 (14)	0.0464 (15)	0.0441 (14)	−0.0024 (13)	0.0079 (12)	0.0175 (13)
N4	0.0385 (15)	0.0663 (19)	0.0591 (17)	−0.0008 (14)	0.0074 (13)	0.0333 (15)
C1	0.0316 (15)	0.0389 (17)	0.0325 (15)	0.0062 (14)	0.0090 (13)	0.0023 (14)
C2	0.0344 (16)	0.0396 (17)	0.0377 (16)	0.0095 (14)	0.0106 (13)	−0.0003 (14)
C3	0.0442 (17)	0.0458 (19)	0.0465 (18)	0.0181 (16)	0.0179 (15)	0.0028 (15)
C4	0.0471 (18)	0.052 (2)	0.0375 (17)	0.0124 (16)	0.0164 (15)	0.0008 (15)
C5	0.0440 (18)	0.0428 (18)	0.0294 (15)	0.0084 (15)	0.0065 (14)	−0.0006 (14)
C6	0.0312 (16)	0.0340 (17)	0.0392 (16)	0.0051 (14)	0.0072 (13)	−0.0050 (14)
C7	0.0375 (16)	0.0336 (16)	0.0331 (16)	0.0081 (14)	0.0000 (14)	−0.0052 (14)
C8	0.0495 (19)	0.0389 (17)	0.0285 (15)	0.0117 (16)	0.0032 (14)	−0.0051 (14)
C9	0.066 (2)	0.0419 (19)	0.0377 (17)	0.0053 (18)	0.0082 (16)	−0.0042 (15)
C10	0.105 (3)	0.046 (2)	0.049 (2)	0.007 (2)	0.026 (2)	0.0054 (17)
C11	0.105 (3)	0.062 (2)	0.041 (2)	0.033 (2)	0.003 (2)	0.0060 (19)
C12	0.077 (3)	0.075 (3)	0.045 (2)	0.034 (2)	−0.009 (2)	−0.003 (2)
C13	0.050 (2)	0.054 (2)	0.0442 (19)	0.0151 (18)	0.0004 (16)	−0.0048 (17)
C14	0.061 (2)	0.072 (2)	0.054 (2)	0.0405 (19)	0.0219 (17)	0.0171 (18)
C15	0.094 (3)	0.085 (3)	0.045 (2)	0.059 (2)	0.0195 (19)	0.0149 (19)
C16	0.0315 (16)	0.0468 (18)	0.0407 (17)	0.0097 (15)	0.0112 (14)	0.0001 (15)
C17	0.0429 (18)	0.051 (2)	0.0375 (16)	0.0212 (16)	0.0149 (14)	0.0071 (15)
C18	0.061 (2)	0.051 (2)	0.062 (2)	0.0161 (19)	0.0267 (18)	0.0044 (18)
C19	0.105 (3)	0.054 (2)	0.071 (2)	0.023 (2)	0.031 (2)	0.005 (2)
C20	0.115 (3)	0.077 (3)	0.066 (2)	0.061 (3)	0.032 (2)	0.010 (2)
C21	0.063 (2)	0.112 (3)	0.072 (3)	0.053 (2)	0.025 (2)	0.008 (2)
C22	0.050 (2)	0.076 (3)	0.061 (2)	0.023 (2)	0.0181 (18)	0.001 (2)
C23	0.084 (3)	0.094 (3)	0.0438 (19)	0.040 (2)	0.0275 (19)	0.006 (2)
C24	0.0306 (16)	0.0436 (18)	0.0364 (16)	−0.0036 (15)	0.0082 (13)	0.0047 (14)
C25	0.0341 (16)	0.0409 (17)	0.0376 (16)	−0.0016 (15)	0.0106 (13)	0.0034 (14)
C26	0.0467 (18)	0.0422 (18)	0.0395 (16)	−0.0061 (15)	0.0196 (14)	0.0023 (14)
C27	0.0346 (16)	0.0431 (18)	0.0406 (17)	−0.0060 (15)	0.0080 (14)	−0.0016 (15)
C28	0.0357 (17)	0.0415 (18)	0.0360 (16)	−0.0005 (15)	0.0027 (14)	−0.0007 (14)
C29	0.0374 (17)	0.0324 (16)	0.0368 (16)	−0.0027 (14)	0.0085 (14)	0.0005 (13)
C30	0.0400 (17)	0.0355 (17)	0.0359 (16)	−0.0073 (15)	0.0029 (14)	0.0010 (14)
C31	0.0374 (18)	0.0356 (17)	0.0480 (19)	−0.0053 (15)	−0.0019 (15)	0.0076 (15)
C32	0.049 (2)	0.0409 (19)	0.060 (2)	−0.0059 (17)	0.0038 (17)	0.0013 (17)
C33	0.052 (2)	0.051 (2)	0.105 (3)	0.008 (2)	0.007 (2)	0.002 (2)
C34	0.058 (2)	0.067 (3)	0.102 (3)	0.009 (2)	−0.015 (2)	0.029 (3)
C35	0.066 (3)	0.077 (3)	0.058 (2)	−0.015 (2)	−0.014 (2)	0.024 (2)
C36	0.051 (2)	0.054 (2)	0.048 (2)	−0.0067 (18)	−0.0026 (17)	0.0053 (17)
C37	0.046 (2)	0.067 (3)	0.040 (3)	0.006 (2)	0.008 (2)	0.024 (2)
C38	0.042 (2)	0.061 (4)	0.076 (4)	0.008 (3)	0.014 (2)	0.035 (3)
C37'	0.046 (2)	0.067 (3)	0.040 (3)	0.006 (2)	0.008 (2)	0.024 (2)
C38'	0.042 (2)	0.061 (4)	0.076 (4)	0.008 (3)	0.014 (2)	0.035 (3)
C39	0.0420 (18)	0.0497 (19)	0.0457 (18)	−0.0010 (16)	0.0190 (15)	0.0100 (15)
C40	0.0364 (17)	0.054 (2)	0.057 (2)	0.0025 (16)	0.0180 (15)	0.0226 (17)
C41	0.066 (2)	0.058 (2)	0.085 (3)	−0.003 (2)	0.028 (2)	0.012 (2)
C42	0.071 (3)	0.055 (3)	0.149 (4)	−0.005 (2)	0.029 (3)	0.020 (3)
C43	0.075 (3)	0.086 (3)	0.140 (4)	0.004 (3)	0.044 (3)	0.064 (3)
C44	0.080 (3)	0.108 (4)	0.085 (3)	0.010 (3)	0.042 (2)	0.048 (3)
C45	0.060 (2)	0.074 (3)	0.067 (2)	0.010 (2)	0.0260 (19)	0.025 (2)
C46	0.0427 (19)	0.066 (2)	0.057 (2)	−0.0076 (18)	0.0131 (17)	0.0036 (18)
C47	0.201 (18)	0.156 (13)	0.35 (3)	0.001 (13)	−0.019 (18)	−0.134 (15)
C48	0.346 (18)	0.231 (17)	0.39 (2)	0.177 (15)	0.275 (16)	0.174 (15)

### Geometric parameters (Å, °)

**Table:**

Cu1—N2	1.920 (3)	C18—H18	0.9300
Cu1—O1^i^	1.9258 (19)	C19—C20	1.349 (6)
Cu1—N1	1.934 (2)	C19—H19	0.9300
Cu1—O1	1.939 (2)	C20—C21	1.350 (6)
Cu1—Cl1	2.4238 (12)	C20—H20	0.9300
Cu1—Cu1^i^	2.9563 (7)	C21—C22	1.377 (6)
Cu2—N4	1.913 (3)	C21—H21	0.9300
Cu2—O2^ii^	1.922 (2)	C22—H22	0.9300
Cu2—N3	1.931 (2)	C23—H23A	0.9600
Cu2—O2	1.943 (2)	C23—H23B	0.9600
Cu2—Cl2	2.4356 (13)	C23—H23C	0.9600
Cu2—Cu2^ii^	2.9512 (8)	C24—C29	1.412 (4)
O1—C1^i^	1.300 (4)	C24—C25^ii^	1.427 (4)
O1—Cu1^i^	1.9258 (19)	C25—C26	1.391 (4)
O2—C24	1.309 (4)	C25—C24^ii^	1.427 (4)
O2—Cu2^ii^	1.922 (2)	C25—C39	1.458 (4)
O3—C48	1.425 (13)	C26—C27	1.372 (4)
O3—H4	0.8200	C26—H26	0.9300
O1W—H1W1	0.820 (17)	C27—C28	1.382 (4)
O1W—H1W2	0.818 (17)	C27—C46	1.500 (4)
O2W—H2W1	0.8494	C28—C29^ii^	1.388 (4)
O2W—H2W2	0.9688	C28—H28	0.9300
O3W—H3W1	1.0487	C29—C28^ii^	1.388 (4)
O3W—H3W2	0.8157	C29—C30	1.485 (4)
N1—C7^i^	1.282 (4)	C30—C31	1.491 (4)
N1—C14	1.467 (4)	C31—C32	1.380 (5)
N2—C16	1.279 (4)	C31—C36	1.386 (5)
N2—C15	1.478 (4)	C32—C33	1.378 (5)
N3—C30	1.279 (4)	C32—H32	0.9300
N3—C37'	1.453 (13)	C33—C34	1.363 (6)
N3—C37	1.502 (6)	C33—H33	0.9300
N4—C39	1.289 (4)	C34—C35	1.363 (6)
N4—C38'	1.458 (12)	C34—H34	0.9300
N4—C38	1.514 (6)	C35—C36	1.380 (5)
C1—O1^i^	1.300 (4)	C35—H35	0.9300
C1—C6	1.413 (4)	C36—H36	0.9300
C1—C2	1.421 (4)	C37—C38	1.517 (7)
C2—C3	1.393 (4)	C37—H37A	0.9700
C2—C16	1.476 (4)	C37—H37B	0.9700
C3—C4	1.366 (4)	C38—H38A	0.9700
C3—H3	0.9300	C38—H38B	0.9700
C4—C5	1.374 (5)	C37'—C38'	1.512 (13)
C4—C23	1.502 (5)	C37'—H37C	0.9700
C5—C6	1.392 (4)	C37'—H37D	0.9700
C5—H5	0.9300	C38'—H38C	0.9700
C6—C7	1.486 (4)	C38'—H38D	0.9700
C7—N1^i^	1.282 (4)	C39—C40	1.490 (5)
C7—C8	1.492 (4)	C40—C41	1.363 (5)
C8—C13	1.384 (4)	C40—C45	1.386 (5)
C8—C9	1.392 (5)	C41—C42	1.389 (6)
C9—C10	1.390 (5)	C41—H41	0.9300
C9—H9	0.9300	C42—C43	1.366 (7)
C10—C11	1.363 (6)	C42—H42	0.9300
C10—H10	0.9300	C43—C44	1.323 (7)
C11—C12	1.348 (6)	C43—H43	0.9300
C11—H11	0.9300	C44—C45	1.382 (6)
C12—C13	1.395 (5)	C44—H44	0.9300
C12—H12	0.9300	C45—H45	0.9300
C13—H13	0.9300	C46—H46A	0.9600
C14—C15	1.452 (5)	C46—H46B	0.9600
C14—H14A	0.9700	C46—H46C	0.9600
C14—H14B	0.9700	C47—C48	1.505 (10)
C15—H15A	0.9700	C47—H47A	0.9600
C15—H15B	0.9700	C47—H47B	0.9600
C16—C17	1.496 (4)	C47—H47C	0.9600
C17—C18	1.354 (5)	C48—H48A	0.9700
C17—C22	1.378 (5)	C48—H48B	0.9700
C18—C19	1.374 (5)		
			
N2—Cu1—O1^i^	90.16 (10)	C18—C19—H19	119.9
N2—Cu1—N1	88.88 (11)	C19—C20—C21	119.9 (4)
O1^i^—Cu1—N1	159.32 (11)	C19—C20—H20	120.0
N2—Cu1—O1	148.23 (11)	C21—C20—H20	120.0
O1^i^—Cu1—O1	80.20 (10)	C20—C21—C22	120.7 (4)
N1—Cu1—O1	89.86 (10)	C20—C21—H21	119.7
N2—Cu1—Cl1	110.60 (9)	C22—C21—H21	119.7
O1^i^—Cu1—Cl1	99.59 (8)	C21—C22—C17	119.4 (4)
N1—Cu1—Cl1	100.07 (8)	C21—C22—H22	120.3
O1—Cu1—Cl1	100.87 (8)	C17—C22—H22	120.3
N2—Cu1—Cu1^i^	124.02 (7)	C4—C23—H23A	109.5
O1^i^—Cu1—Cu1^i^	40.26 (7)	C4—C23—H23B	109.5
N1—Cu1—Cu1^i^	127.43 (8)	H23A—C23—H23B	109.5
O1—Cu1—Cu1^i^	39.93 (6)	C4—C23—H23C	109.5
Cl1—Cu1—Cu1^i^	103.43 (4)	H23A—C23—H23C	109.5
N4—Cu2—O2^ii^	90.49 (10)	H23B—C23—H23C	109.5
N4—Cu2—N3	88.75 (11)	O2—C24—C29	120.3 (3)
O2^ii^—Cu2—N3	159.37 (11)	O2—C24—C25^ii^	119.8 (3)
N4—Cu2—O2	147.45 (12)	C29—C24—C25^ii^	119.9 (3)
O2^ii^—Cu2—O2	80.47 (10)	C26—C25—C24^ii^	117.5 (3)
N3—Cu2—O2	89.18 (10)	C26—C25—C39	118.2 (3)
N4—Cu2—Cl2	110.11 (10)	C24^ii^—C25—C39	124.2 (3)
O2^ii^—Cu2—Cl2	99.31 (8)	C27—C26—C25	123.9 (3)
N3—Cu2—Cl2	100.31 (9)	C27—C26—H26	118.1
O2—Cu2—Cl2	102.22 (8)	C25—C26—H26	118.1
N4—Cu2—Cu2^ii^	124.09 (8)	C26—C27—C28	117.2 (3)
O2^ii^—Cu2—Cu2^ii^	40.49 (7)	C26—C27—C46	121.1 (3)
N3—Cu2—Cu2^ii^	126.88 (8)	C28—C27—C46	121.6 (3)
O2—Cu2—Cu2^ii^	39.97 (6)	C27—C28—C29^ii^	123.4 (3)
Cl2—Cu2—Cu2^ii^	104.16 (4)	C27—C28—H28	118.3
C1^i^—O1—Cu1^i^	131.06 (19)	C29^ii^—C28—H28	118.3
C1^i^—O1—Cu1	127.82 (18)	C28^ii^—C29—C24	118.2 (3)
Cu1^i^—O1—Cu1	99.80 (10)	C28^ii^—C29—C30	118.2 (3)
C24—O2—Cu2^ii^	131.45 (19)	C24—C29—C30	123.6 (3)
C24—O2—Cu2	126.42 (19)	N3—C30—C29	121.6 (3)
Cu2^ii^—O2—Cu2	99.54 (10)	N3—C30—C31	121.3 (3)
C48—O3—H4	109.5	C29—C30—C31	117.1 (3)
H1W1—O1W—H1W2	105 (3)	C32—C31—C36	118.7 (3)
H2W1—O2W—H2W2	102.3	C32—C31—C30	119.5 (3)
H3W1—O3W—H3W2	116.8	C36—C31—C30	121.7 (3)
C7^i^—N1—C14	124.5 (3)	C33—C32—C31	120.4 (4)
C7^i^—N1—Cu1	128.4 (2)	C33—C32—H32	119.8
C14—N1—Cu1	107.0 (2)	C31—C32—H32	119.8
C16—N2—C15	120.4 (3)	C34—C33—C32	120.4 (4)
C16—N2—Cu1	129.1 (2)	C34—C33—H33	119.8
C15—N2—Cu1	109.0 (2)	C32—C33—H33	119.8
C30—N3—C37'	122.1 (5)	C35—C34—C33	119.9 (4)
C30—N3—C37	123.6 (3)	C35—C34—H34	120.0
C30—N3—Cu2	128.9 (2)	C33—C34—H34	120.0
C37'—N3—Cu2	106.2 (4)	C34—C35—C36	120.5 (4)
C37—N3—Cu2	107.4 (2)	C34—C35—H35	119.7
C39—N4—C38'	124.2 (5)	C36—C35—H35	119.7
C39—N4—C38	118.2 (3)	C35—C36—C31	120.0 (4)
C39—N4—Cu2	129.2 (2)	C35—C36—H36	120.0
C38'—N4—Cu2	105.7 (5)	C31—C36—H36	120.0
C38—N4—Cu2	109.5 (3)	N3—C37—C38	107.5 (4)
O1^i^—C1—C6	120.4 (3)	N3—C37—H37A	110.2
O1^i^—C1—C2	120.2 (2)	C38—C37—H37A	110.2
C6—C1—C2	119.3 (3)	N3—C37—H37B	110.2
C3—C2—C1	118.0 (3)	C38—C37—H37B	110.2
C3—C2—C16	118.4 (3)	H37A—C37—H37B	108.5
C1—C2—C16	123.4 (3)	N4—C38—C37	108.1 (4)
C4—C3—C2	123.6 (3)	N4—C38—H38A	110.1
C4—C3—H3	118.2	C37—C38—H38A	110.1
C2—C3—H3	118.2	N4—C38—H38B	110.1
C3—C4—C5	117.4 (3)	C37—C38—H38B	110.1
C3—C4—C23	121.8 (3)	H38A—C38—H38B	108.4
C5—C4—C23	120.8 (3)	N3—C37'—C38'	110.5 (10)
C4—C5—C6	123.4 (3)	N3—C37'—H37C	109.6
C4—C5—H5	118.3	C38'—C37'—H37C	109.6
C6—C5—H5	118.3	N3—C37'—H37D	109.6
C5—C6—C1	118.3 (3)	C38'—C37'—H37D	109.6
C5—C6—C7	117.8 (3)	H37C—C37'—H37D	108.1
C1—C6—C7	123.9 (3)	N4—C38'—C37'	104.8 (9)
N1^i^—C7—C6	122.2 (3)	N4—C38'—H38C	110.8
N1^i^—C7—C8	120.6 (3)	C37'—C38'—H38C	110.8
C6—C7—C8	117.1 (3)	N4—C38'—H38D	110.8
C13—C8—C9	118.8 (3)	C37'—C38'—H38D	110.8
C13—C8—C7	121.5 (3)	H38C—C38'—H38D	108.9
C9—C8—C7	119.6 (3)	N4—C39—C25	123.1 (3)
C10—C9—C8	120.0 (3)	N4—C39—C40	118.8 (3)
C10—C9—H9	120.0	C25—C39—C40	118.1 (3)
C8—C9—H9	120.0	C41—C40—C45	118.9 (3)
C11—C10—C9	120.1 (4)	C41—C40—C39	121.5 (3)
C11—C10—H10	120.0	C45—C40—C39	119.6 (3)
C9—C10—H10	120.0	C40—C41—C42	119.9 (4)
C12—C11—C10	120.6 (4)	C40—C41—H41	120.0
C12—C11—H11	119.7	C42—C41—H41	120.0
C10—C11—H11	119.7	C43—C42—C41	119.8 (5)
C11—C12—C13	120.7 (4)	C43—C42—H42	120.1
C11—C12—H12	119.7	C41—C42—H42	120.1
C13—C12—H12	119.7	C44—C43—C42	120.7 (4)
C8—C13—C12	119.8 (4)	C44—C43—H43	119.6
C8—C13—H13	120.1	C42—C43—H43	119.6
C12—C13—H13	120.1	C43—C44—C45	120.7 (5)
C15—C14—N1	112.2 (3)	C43—C44—H44	119.7
C15—C14—H14A	109.2	C45—C44—H44	119.7
N1—C14—H14A	109.2	C44—C45—C40	119.9 (4)
C15—C14—H14B	109.2	C44—C45—H45	120.0
N1—C14—H14B	109.2	C40—C45—H45	120.0
H14A—C14—H14B	107.9	C27—C46—H46A	109.5
C14—C15—N2	112.2 (3)	C27—C46—H46B	109.5
C14—C15—H15A	109.2	H46A—C46—H46B	109.5
N2—C15—H15A	109.2	C27—C46—H46C	109.5
C14—C15—H15B	109.2	H46A—C46—H46C	109.5
N2—C15—H15B	109.2	H46B—C46—H46C	109.5
H15A—C15—H15B	107.9	C48—C47—H47A	109.5
N2—C16—C2	122.8 (3)	C48—C47—H47B	109.5
N2—C16—C17	119.2 (3)	H47A—C47—H47B	109.5
C2—C16—C17	117.9 (3)	C48—C47—H47C	109.5
C18—C17—C22	119.2 (3)	H47A—C47—H47C	109.5
C18—C17—C16	120.6 (3)	H47B—C47—H47C	109.5
C22—C17—C16	120.0 (3)	O3—C48—C47	106.2 (9)
C17—C18—C19	120.5 (4)	O3—C48—H48A	110.5
C17—C18—H18	119.7	C47—C48—H48A	110.5
C19—C18—H18	119.7	O3—C48—H48B	110.5
C20—C19—C18	120.3 (4)	C47—C48—H48B	110.5
C20—C19—H19	119.9	H48A—C48—H48B	108.7
			
N2—Cu1—O1—C1^i^	117.9 (3)	C11—C12—C13—C8	−0.3 (6)
O1^i^—Cu1—O1—C1^i^	−168.0 (3)	C7^i^—N1—C14—C15	146.7 (3)
N1—Cu1—O1—C1^i^	30.2 (3)	Cu1—N1—C14—C15	−32.8 (4)
Cl1—Cu1—O1—C1^i^	−70.0 (3)	N1—C14—C15—N2	35.9 (5)
Cu1^i^—Cu1—O1—C1^i^	−168.0 (3)	C16—N2—C15—C14	172.2 (3)
N2—Cu1—O1—Cu1^i^	−74.1 (2)	Cu1—N2—C15—C14	−20.5 (4)
O1^i^—Cu1—O1—Cu1^i^	0.0	C15—N2—C16—C2	169.5 (3)
N1—Cu1—O1—Cu1^i^	−161.78 (11)	Cu1—N2—C16—C2	5.0 (5)
Cl1—Cu1—O1—Cu1^i^	97.99 (9)	C15—N2—C16—C17	−7.4 (5)
N4—Cu2—O2—C24	−121.0 (3)	Cu1—N2—C16—C17	−171.8 (2)
O2^ii^—Cu2—O2—C24	163.3 (3)	C3—C2—C16—N2	169.0 (3)
N3—Cu2—O2—C24	−34.6 (3)	C1—C2—C16—N2	−15.4 (5)
Cl2—Cu2—O2—C24	65.8 (3)	C3—C2—C16—C17	−14.1 (4)
Cu2^ii^—Cu2—O2—C24	163.3 (3)	C1—C2—C16—C17	161.5 (3)
N4—Cu2—O2—Cu2^ii^	75.7 (2)	N2—C16—C17—C18	95.3 (4)
O2^ii^—Cu2—O2—Cu2^ii^	0.0	C2—C16—C17—C18	−81.7 (4)
N3—Cu2—O2—Cu2^ii^	162.08 (12)	N2—C16—C17—C22	−80.7 (4)
Cl2—Cu2—O2—Cu2^ii^	−97.55 (9)	C2—C16—C17—C22	102.3 (4)
N2—Cu1—N1—C7^i^	−162.5 (3)	C22—C17—C18—C19	1.7 (5)
O1^i^—Cu1—N1—C7^i^	−75.0 (4)	C16—C17—C18—C19	−174.4 (3)
O1—Cu1—N1—C7^i^	−14.2 (3)	C17—C18—C19—C20	−1.2 (6)
Cl1—Cu1—N1—C7^i^	86.8 (3)	C18—C19—C20—C21	−0.4 (6)
Cu1^i^—Cu1—N1—C7^i^	−28.9 (3)	C19—C20—C21—C22	1.3 (7)
N2—Cu1—N1—C14	17.0 (2)	C20—C21—C22—C17	−0.7 (6)
O1^i^—Cu1—N1—C14	104.5 (3)	C18—C17—C22—C21	−0.8 (5)
O1—Cu1—N1—C14	165.2 (2)	C16—C17—C22—C21	175.3 (3)
Cl1—Cu1—N1—C14	−93.8 (2)	Cu2^ii^—O2—C24—C29	−170.8 (2)
Cu1^i^—Cu1—N1—C14	150.57 (19)	Cu2—O2—C24—C29	31.4 (4)
O1^i^—Cu1—N2—C16	8.0 (3)	Cu2^ii^—O2—C24—C25^ii^	10.0 (5)
N1—Cu1—N2—C16	167.4 (3)	Cu2—O2—C24—C25^ii^	−147.8 (2)
O1—Cu1—N2—C16	79.5 (4)	C24^ii^—C25—C26—C27	−0.5 (5)
Cl1—Cu1—N2—C16	−92.3 (3)	C39—C25—C26—C27	−175.7 (3)
Cu1^i^—Cu1—N2—C16	31.3 (3)	C25—C26—C27—C28	0.5 (5)
O1^i^—Cu1—N2—C15	−157.8 (2)	C25—C26—C27—C46	177.1 (3)
N1—Cu1—N2—C15	1.5 (2)	C26—C27—C28—C29^ii^	0.3 (5)
O1—Cu1—N2—C15	−86.4 (3)	C46—C27—C28—C29^ii^	−176.2 (3)
Cl1—Cu1—N2—C15	101.9 (2)	O2—C24—C29—C28^ii^	179.7 (3)
Cu1^i^—Cu1—N2—C15	−134.6 (2)	C25^ii^—C24—C29—C28^ii^	−1.1 (5)
N4—Cu2—N3—C30	164.0 (3)	O2—C24—C29—C30	−0.9 (5)
O2^ii^—Cu2—N3—C30	75.9 (4)	C25^ii^—C24—C29—C30	178.3 (3)
O2—Cu2—N3—C30	16.4 (3)	C37'—N3—C30—C29	161.7 (6)
Cl2—Cu2—N3—C30	−85.8 (3)	C37—N3—C30—C29	−172.6 (3)
Cu2^ii^—Cu2—N3—C30	30.8 (3)	Cu2—N3—C30—C29	3.5 (4)
N4—Cu2—N3—C37'	3.1 (6)	C37'—N3—C30—C31	−17.9 (7)
O2^ii^—Cu2—N3—C37'	−85.0 (6)	C37—N3—C30—C31	7.9 (5)
O2—Cu2—N3—C37'	−144.4 (6)	Cu2—N3—C30—C31	−176.1 (2)
Cl2—Cu2—N3—C37'	113.3 (6)	C28^ii^—C29—C30—N3	162.3 (3)
Cu2^ii^—Cu2—N3—C37'	−130.1 (6)	C24—C29—C30—N3	−17.1 (5)
N4—Cu2—N3—C37	−19.5 (3)	C28^ii^—C29—C30—C31	−18.1 (4)
O2^ii^—Cu2—N3—C37	−107.6 (4)	C24—C29—C30—C31	162.5 (3)
O2—Cu2—N3—C37	−167.0 (3)	N3—C30—C31—C32	121.2 (4)
Cl2—Cu2—N3—C37	90.7 (3)	C29—C30—C31—C32	−58.4 (4)
Cu2^ii^—Cu2—N3—C37	−152.7 (2)	N3—C30—C31—C36	−56.2 (4)
O2^ii^—Cu2—N4—C39	−6.0 (3)	C29—C30—C31—C36	124.2 (3)
N3—Cu2—N4—C39	−165.3 (3)	C36—C31—C32—C33	2.3 (5)
O2—Cu2—N4—C39	−78.8 (4)	C30—C31—C32—C33	−175.2 (3)
Cl2—Cu2—N4—C39	94.1 (3)	C31—C32—C33—C34	−1.7 (6)
Cu2^ii^—Cu2—N4—C39	−30.1 (4)	C32—C33—C34—C35	0.2 (6)
O2^ii^—Cu2—N4—C38'	−175.0 (6)	C33—C34—C35—C36	0.7 (6)
N3—Cu2—N4—C38'	25.6 (6)	C34—C35—C36—C31	0.0 (6)
O2—Cu2—N4—C38'	112.1 (6)	C32—C31—C36—C35	−1.5 (5)
Cl2—Cu2—N4—C38'	−74.9 (6)	C30—C31—C36—C35	176.0 (3)
Cu2^ii^—Cu2—N4—C38'	160.9 (6)	C30—N3—C37—C38	−142.6 (4)
O2^ii^—Cu2—N4—C38	153.1 (3)	C37'—N3—C37—C38	−49.3 (12)
N3—Cu2—N4—C38	−6.2 (3)	Cu2—N3—C37—C38	40.7 (5)
O2—Cu2—N4—C38	80.3 (3)	C39—N4—C38—C37	−167.8 (4)
Cl2—Cu2—N4—C38	−106.8 (3)	C38'—N4—C38—C37	−57.4 (10)
Cu2^ii^—Cu2—N4—C38	129.0 (3)	Cu2—N4—C38—C37	30.5 (5)
O1^i^—C1—C2—C3	−178.2 (3)	N3—C37—C38—N4	−46.5 (6)
C6—C1—C2—C3	2.5 (4)	C30—N3—C37'—C38'	166.6 (7)
O1^i^—C1—C2—C16	6.2 (5)	C37—N3—C37'—C38'	65.7 (14)
C6—C1—C2—C16	−173.1 (3)	Cu2—N3—C37'—C38'	−30.9 (10)
C1—C2—C3—C4	−1.0 (5)	C39—N4—C38'—C37'	143.6 (8)
C16—C2—C3—C4	174.8 (3)	C38—N4—C38'—C37'	55.2 (10)
C2—C3—C4—C5	−0.9 (5)	Cu2—N4—C38'—C37'	−46.7 (10)
C2—C3—C4—C23	−179.2 (3)	N3—C37'—C38'—N4	52.5 (12)
C3—C4—C5—C6	1.3 (5)	C38'—N4—C39—C25	163.2 (7)
C23—C4—C5—C6	179.7 (3)	C38—N4—C39—C25	−161.6 (4)
C4—C5—C6—C1	0.2 (5)	Cu2—N4—C39—C25	−4.0 (5)
C4—C5—C6—C7	−179.0 (3)	C38'—N4—C39—C40	−19.2 (8)
O1^i^—C1—C6—C5	178.6 (3)	C38—N4—C39—C40	16.0 (5)
C2—C1—C6—C5	−2.1 (4)	Cu2—N4—C39—C40	173.6 (2)
O1^i^—C1—C6—C7	−2.3 (5)	C26—C25—C39—N4	−173.4 (3)
C2—C1—C6—C7	177.0 (3)	C24^ii^—C25—C39—N4	11.7 (5)
C5—C6—C7—N1^i^	165.9 (3)	C26—C25—C39—C40	9.0 (5)
C1—C6—C7—N1^i^	−13.2 (5)	C24^ii^—C25—C39—C40	−165.9 (3)
C5—C6—C7—C8	−15.8 (4)	N4—C39—C40—C41	−95.9 (4)
C1—C6—C7—C8	165.1 (3)	C25—C39—C40—C41	81.8 (4)
N1^i^—C7—C8—C13	−55.5 (4)	N4—C39—C40—C45	83.3 (4)
C6—C7—C8—C13	126.2 (3)	C25—C39—C40—C45	−99.0 (4)
N1^i^—C7—C8—C9	120.7 (3)	C45—C40—C41—C42	−1.5 (5)
C6—C7—C8—C9	−57.6 (4)	C39—C40—C41—C42	177.6 (3)
C13—C8—C9—C10	2.4 (5)	C40—C41—C42—C43	2.5 (6)
C7—C8—C9—C10	−173.9 (3)	C41—C42—C43—C44	−2.3 (7)
C8—C9—C10—C11	−1.6 (5)	C42—C43—C44—C45	1.2 (7)
C9—C10—C11—C12	−0.2 (6)	C43—C44—C45—C40	−0.2 (6)
C10—C11—C12—C13	1.1 (6)	C41—C40—C45—C44	0.4 (5)
C9—C8—C13—C12	−1.5 (5)	C39—C40—C45—C44	−178.8 (3)
C7—C8—C13—C12	174.7 (3)		

Symmetry codes: (i) −*x*+2, −*y*+1, −*z*+2; (ii) −*x*+1, −*y*+1, −*z*+1.

### Hydrogen-bond geometry (Å, °)

**Table:**

*D*—H···*A*	*D*—H	H···*A*	*D*···*A*	*D*—H···*A*
O3—H4···O2W	0.82	2.20	2.96 (2)	154
O1W—H1W1···Cl1^iii^	0.82 (2)	2.54 (2)	3.355 (4)	171 (4)
O1W—H1W2···Cl2	0.82 (2)	2.56 (2)	3.374 (4)	174 (3)
O2W—H2W1···O3W	0.85	1.86	2.71 (3)	179
O2W—H2W2···O3	0.97	2.27	2.96 (2)	127

Symmetry codes: (iii) *x*−1/2, −*y*+3/2, *z*−1/2.
